# Epithelial cyst arising in an intrapancreatic accessory spleen: a case report of robotic surgery and review of minimally invasive treatment

**DOI:** 10.1186/s12893-020-00927-0

**Published:** 2020-10-31

**Authors:** Tomokatsu Kato, Yoichi Matsuo, Goro Ueda, Yoshinaga Aoyama, Kan Omi, Yuichi Hayashi, Hiroyuki Imafuji, Kenta Saito, Ken Tsuboi, Mamoru Morimoto, Ryo Ogawa, Hiroki Takahashi, Hiroyuki Kato, Michihiro Yoshida, Itaru Naitoh, Kazuki Hayashi, Satoru Takahashi, Shuji Takiguchi

**Affiliations:** 1 Department of Gastroenterological Surgery, Nagoya City University Graduate School of Medical Sciences, Kawasumi 1, Mizuho-cho, Mizuho-ku, Nagoya, 4678601 Japan; 2 Department of Experimental Pathology and Tumor Biology, Nagoya City University Graduate School of Medical Sciences, Nagoya, Japan; 3 Department of Gastroenterology and Metabolism, Nagoya City University Graduate School of Medical Sciences, Nagoya, Japan

**Keywords:** Epithelial cyst in an intrapancreatic accessory spleen (ECIPAS), Minimally invasive surgery, Robot-assisted surgery

## Abstract

**Background:**

An epithelial cyst in an intrapancreatic accessory spleen (ECIPAS) is rare. We report a case of ECIPAS that was treated with robot-assisted distal pancreatectomy with splenectomy.

**Case presentation:**

The case was a 59-year-old woman who was referred to our hospital after a pancreatic tail tumor was found on computed tomography prior to surgery for small bowel obstruction at another hospital. A cystic lesion in the pancreatic tail was discovered and evaluated by magnetic resonance imaging and endoscopic ultrasonography. Based on clinical and radiological features, mucinous cystic neoplasm was included in the differential diagnosis. The patient underwent robot-assisted distal pancreatectomy with splenectomy. The postoperative course was uneventful. Pathological evaluation revealed a 20-mm ECIPAS in the pancreatic tail.

**Conclusions:**

If a pancreatic tail tumor is present, ECIPAS should be included in the differential diagnosis. However, preoperative diagnosis is difficult, and a definitive diagnosis is often not obtained until after surgery. Surgery should be minimally invasive. Laparoscopic distal pancreatectomy has become a standard surgical procedure because it is minimally invasive. Robot-assisted surgery is not only minimally invasive, but also advantageous, because it has a stereoscopic magnifying effect and allows the forceps to move smoothly. Robot-assisted distal pancreatectomy may be a good option, when performing surgery for a pancreatic tail tumor.

## Background

An accessory spleen is not rare, as it is observed in 10% of patients at necropsy [1]. Of the accessory spleens identified, 80% are located in the splenic hilum, and 17% are located within the pancreatic tail [2]. However, occurrence of an epithelial cyst in an intrapancreatic accessory spleen (ECIPAS) is rare. ECIPAS is a multilocular or single tufted cystic lesion of the pancreas covered with stratiform squamous epithelium, transitional epithelium or stratified cuboidal epithelium, the cyst of which is surrounded by splenic tissue. Some have been reported as epidermoid cyst. It is a type of epithelial cyst that is covered only by squamous and transitional epithelium without skin appendages [3]. Although the number of reported cases has been increasing in recent years, it is difficult to diagnose preoperatively using conventional imaging such as ultrasonography (US), computed tomography (CT), and magnetic resonance imaging (MRI). ECIPAS is a benign disease, and does not require surgical resection, but it is often misdiagnosed as a cystic neuroendocrine tumor or a solid pseudopapillary tumor, and most patients with ECIPAS undergo surgical resection. If a cystic tumor is found in the tail of the pancreas, ECIPAS should be considered. When surgery is required, it should be minimally invasive, for example, laparoscopic or robotic surgery. Here we report a case of a patient with ECIPAS who underwent robot-assisted distal pancreatectomy with splenectomy.

## Case presentation

**Fig. 1:**
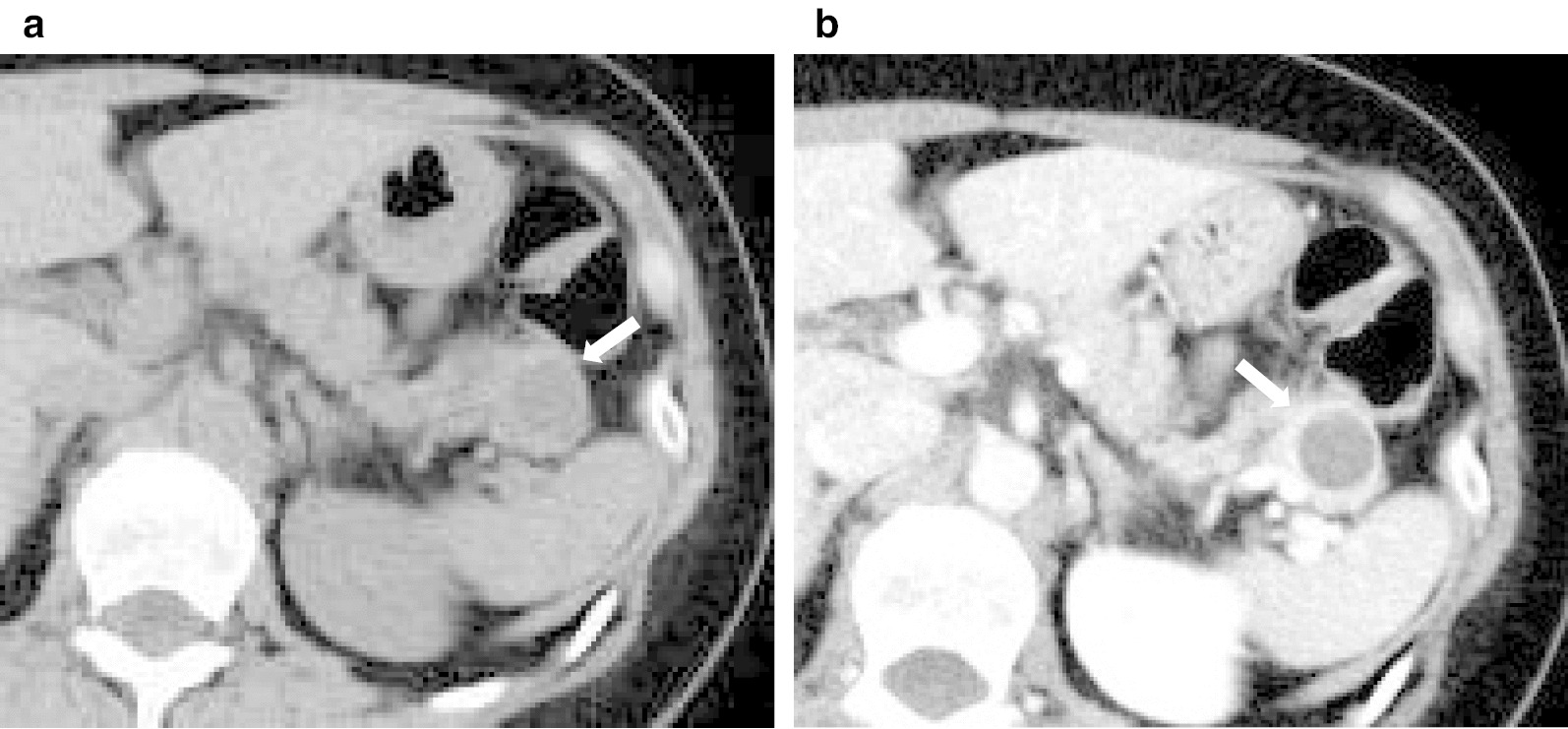
The contrast-enhanced abdominal computed tomography (CT) scan confirmed a unilocular cystic lesion (arrows) measuring 16 mm in size in the pancreatic tail. The wall of the cyst appeared to be enhanced. (**a** plain, **b** portal phase)

**Fig. 2:**
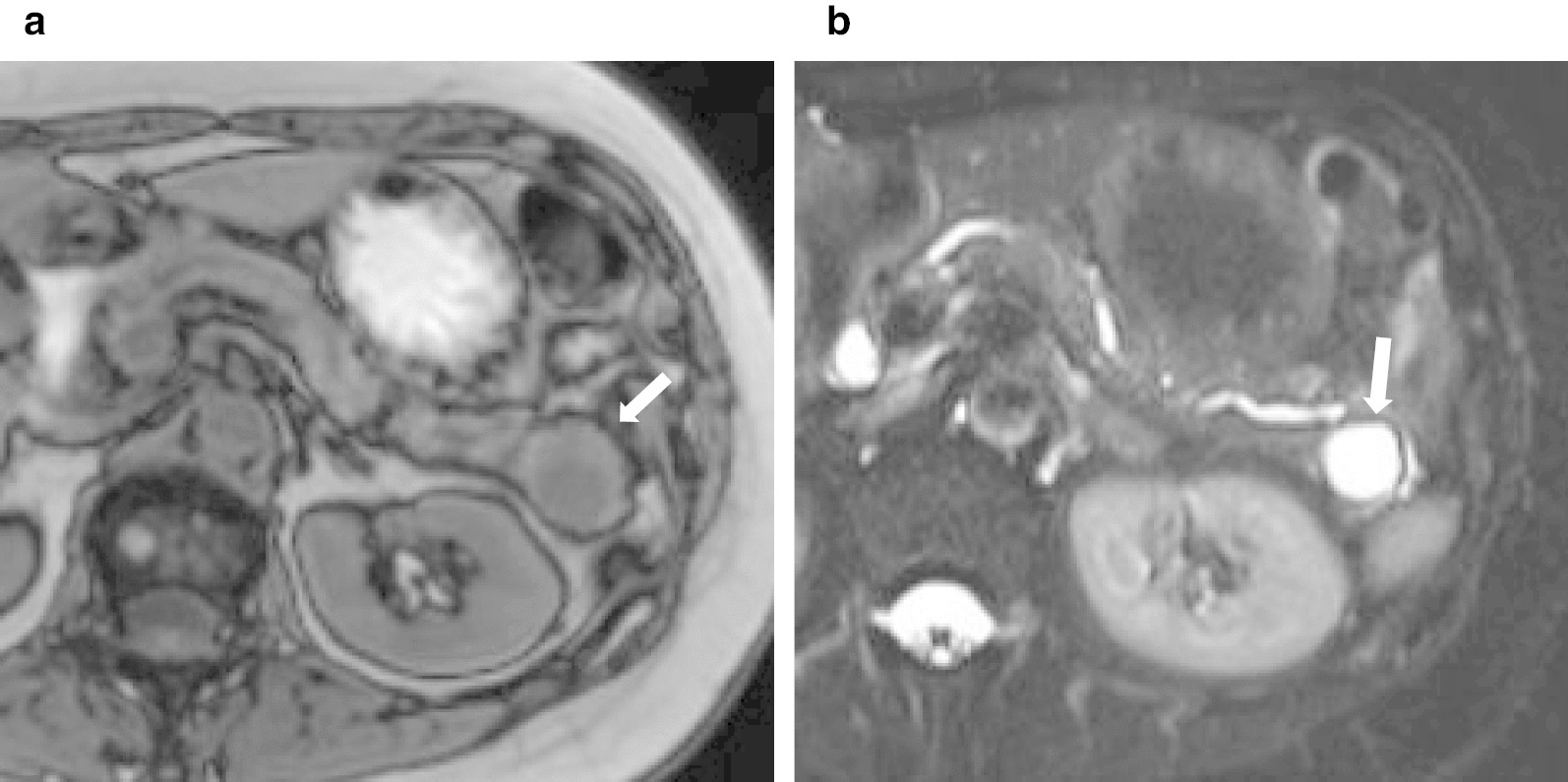
Magnetic resonance images (MRI) revealed that the cystic component showed a low signal intensity (arrow) on T1-weighted images (**a**) and a high signal intensity (arrow) on T2-weighted images (**b**)

**Fig. 3:**
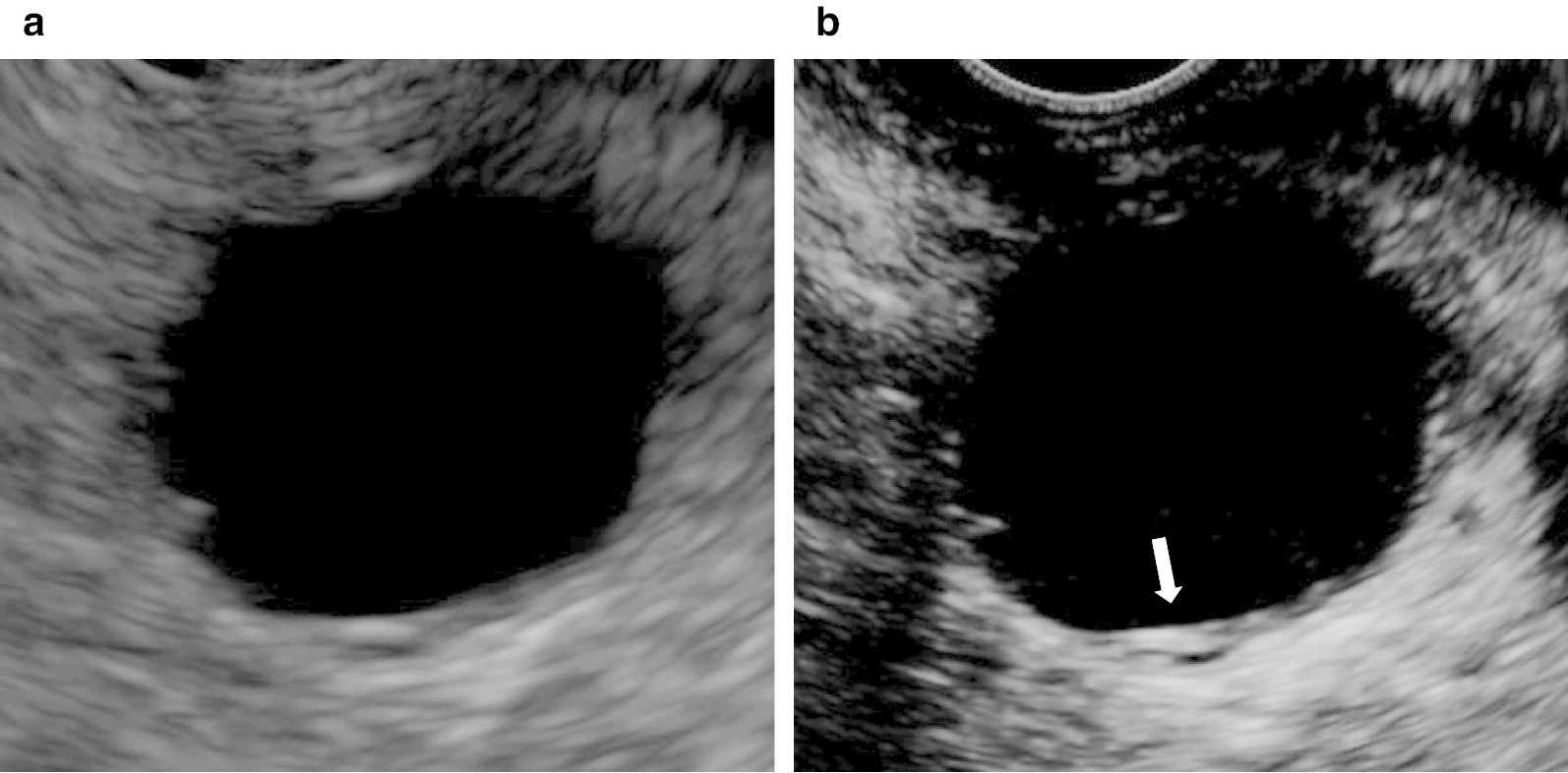
Endoscopic ultrasonography (EUS) showed a unilocular cystic lesion with a partial thickened wall in the pancreatic tail (**a**). The thickened wall was enhanced (arrow) when Sonazoid was administered (**b**)

**Fig. 4:**
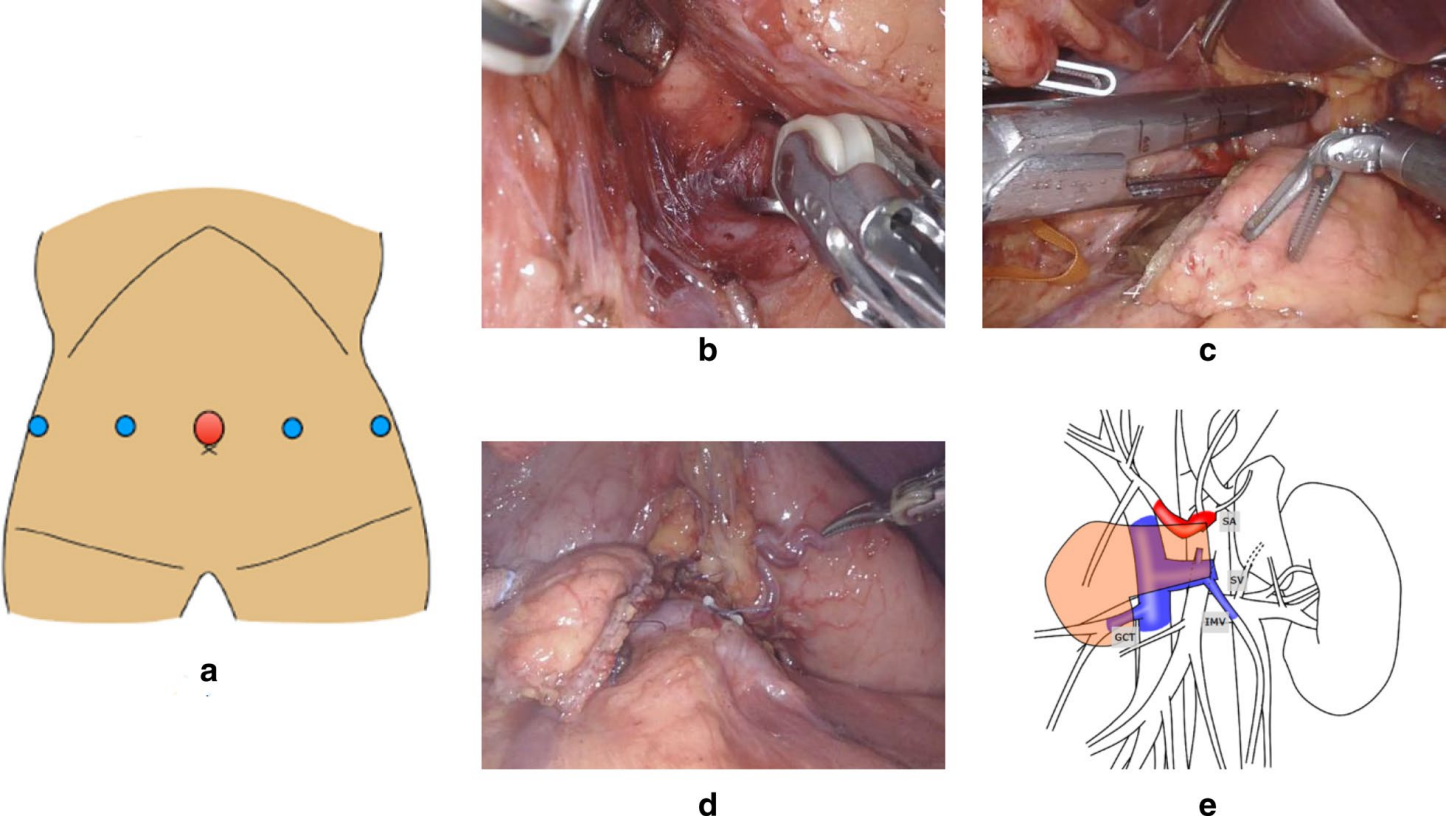
**a** Port placement of robot-assisted distal pancreatectomy. **b** Detachment of the posterior surface of the pancreas and the anterior surface of the superior mesenteric vein (SMV). **c** The pancreas was transected with a stapler device. **d** State after the resection. **e** Sketch after distal pancreatectomy. Figure was created by ourselves

A 59-year-old woman was referred to our hospital after a pancreatic tail tumor was detected by CT prior to surgery for small bowel obstruction at another hospital. She was asymptomatic, and a physical examination revealed no remarkable abnormalities. A laboratory examination showed normal findings. The tumor markers CA19-9 (34.6 U/mL; normal range, < 37 U/mL) and CEA (0.8 ng/mL; normal range, < 5.0 ng/mL) were within the normal range. Enhanced-contrast abdominal CT showed a unilocular cystic lesion measuring 16 mm in size in the pancreatic tail (Fig. 1). The wall of the cyst appeared to be enhanced. MRI showed that the cystic lesion exhibited low intensity on T1-weighted images and high intensity on T2-weighted images (Fig. 2). Endoscopic ultrasonography (EUS) showed a unilocular cystic lesion with a partial thickened wall in the pancreatic tail (Fig. 3a). Contrast-enhanced harmonic EUS with Sonazoid showed that the thickened wall was enhanced (Fig. 3b). The cystic lesion did not communicate with the main pancreatic duct. Given that cystic neoplasms including mucinous cystic neoplasms (MCN) were included in the differential diagnosis, robot-assisted distal pancreatectomy with splenectomy was planned as a minimally invasive method for both diagnostic and treatment purposes. Robot-assisted distal pancreatectomy with splenectomy was performed with the da Vinci system (Fig. 4). Taking sufficient margins, we transected the pancreatic tail with a stapler device and extracted it from the abdominal cavity in an endobag. The total operation time was 288 min, with a total blood loss of 161 ml.

**Fig. 5:**
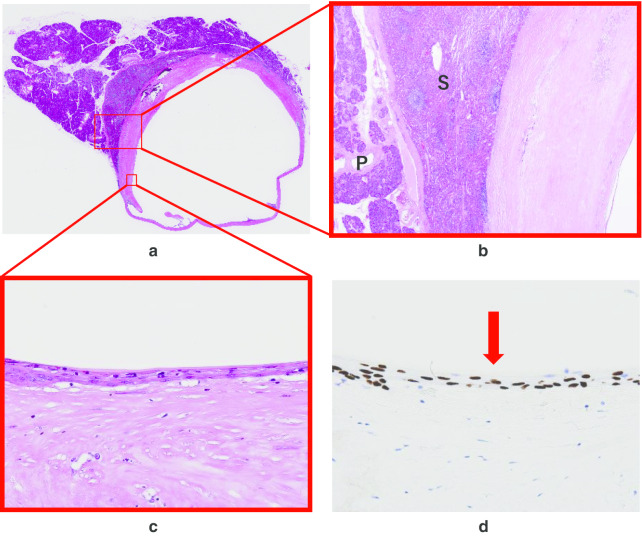
Pathologic specimens. **a** Hematoxylin and eosin (H&E) staining, loupe magnification, **b** pancreatic parenchyma (P) and adjacent splenic parenchyma (S), (H&E staining, × 20), **c** the cyst wall lined by epithelium without atypia, (H&E staining, × 200), **d** p40 staining showing positivity (arrow) in the cyst lining (× 200)

Macroscopically, a cystic lesion with a small cyst was found with a septum with a diameter of 20 mm at the tail of the pancreas. Histologically, the cyst wall consisted of splenic tissue (Fig. 5b). The inner surface of the cyst wall was covered with squamous epithelium (Fig. 5c). The epithelium was positive for CKAE1/3, p40 (Fig. 5d) and CK5/6. Based on these findings, ECIPAS was established as the final pathological diagnosis. The patient was discharged on the 14th postoperative day after an uneventful postoperative course.

## Discussion and conclusions

ECIPAS was first reported in 1980 by Davidson et al. [4] Since then, a total of 59 cases have been reported in the English-language literature [4–42]. According to Li et al. [43] ECIPAS is more common in women, with all cases located in the pancreatic tail, and more than half of the cases were asymptomatic and found incidentally.

Accurate preoperative diagnosis of pancreatic cystic tumors is required. The individual component of ECIPAS shows the same echo image as the spleen. On contrast-enhanced CT, the cyst wall of ECIPAS shows contrast enhancement similar to that of the spleen [29]. On MRI, individual components of ECIPAS show the same signal intensity as the spleen, and cystic lesions generally show low signal intensity on T1-weighted images and high signal intensity on T2-weighted images. It has been reported that endoscopic ultrasound-fine needle aspiration (EUS-FNA) is useful for diagnosing ECIPAS [44–47]. However, accurate preoperative diagnosis is difficult when the amount of accessory spleen tissue is small.

When a pancreatic tail cyst tumor is found, it is important to diagnose with ECIPAS in mind, but a pancreatic malignant cystic tumor cannot be ruled out, and surgery may be performed. In recent years, minimally invasive surgery, such as laparoscopic surgery and robot-assisted surgery, has been adopted for various diseases. Minimally invasive surgery is useful to avoid the disadvantages of open surgery, such as increased pain and prolonged hospitalization. Minimally invasive surgery is recommended for pancreatic tail cystic tumors with suspected ECIPAS.

Since Itano et al. [20] first reported laparoscopic surgery for ECIPAS, there have been 14 cases involving minimally invasive surgery (Table 1) [24–26, 28, 30, 33, 35–37, 39, 40, 42]. In these cases, the median operation time was 203.5 min, median blood loss was 50 mL, and median hospital stay was 12 days. There was only one complication, a Grade A pancreatic fistula.

**Table 1:** Summary of all cases of ECIPAS treated with minimally invasive surgery

No	First author, year	Age, year	Sex	Symptom	Size, cm	Preoperative diagnosis	Surgical procedure	Operative time, min	Intraoperative blood loss, ml	Postoperative complications	Postoperative hospital stay, day
1	Itano 2010	67	M	Epigastric pain	1.5	ECIPAS	LDP	227	400	None	7
2	Khashab 2011	49	F	Abdominal pain	2.3	NET	LSPDP	ND	ND	ND	ND
3	Iwasaki 2011	36	F	Left hypochondalgia	3.4	MCN	LDP	180	30	None	12
4	Urakami 2011	50	F	Asymptomatic	3.0	ECIPAS	LSPDP	246	Minimal	None	10
5	Panagitopoulos 2012	51	M	Asymptomatic	2.0	Malignant cystic tumor	LSPDP	ND	ND	None	3
6	Harris 2012	39	M	Asymptomatic	2.5	Malignant cystic tumor	LDP	140	250	None	8
7	Wakasugi 2013	37	F	Asymptomatic	4.0	MCN, IPMN	LDP	278	50	PF(grade A)	21
8	Kwak 2016	21	F	Abdominal pain, fever	2.5	SPN	LDP	ND	ND	ND	ND
9	Fujii 2016	50	F	Asymptomatic	5.0	MCN	LSPDP	ND	ND	None	14
10	Fujii 2016	60	F	Back discomfort	3.5	IPMN	LDP	ND	ND	None	14
11	van Dijck 2016	21	F	Abdominal pain	2.6	MCA, NET	RSPDP	124	20	None	5
12	Kato 2016	33	F	Asymptomatic	3.0	SPN, NET	LSPDP	ND	ND	ND	ND
13	Suzumura 2017	57	F	Asymptomatic	2.2	MCN	LSPDP	144	10	None	12
14	Paredes 2018	17	F	nausea	3.6	MCN, IPMN	RDP	ND	ND	None	3
15	Our case 2020	59	F	Asymptomatic	2.0	MCN	RDP	288	161	None	14

*ND* not described, *M* male, *F* female, *ECIPAS* epithelial cyst in an intrapancreatic accessory spleen, *NET* neuroendocrine tumor, *MCN* mucinous cystic neoplasm, *IPMN* intraductal papillary mucinous neoplasm, *SPN* solid pseudopapillary neoplasm, *LDP* laparoscopic distal pancreatectomy, *LSPDP* laparoscopic spleen preserving distal pancreatectomy, *RDP* robot-assisted distal pancreatectomy, *RSPDP* robot-assisted spleen preserving distal pancreatectomy, *PF* pancreatic fistula

Among minimally invasive surgical techniques for ECIPAS, robot-assisted surgery has recently become popular. Since van Dijck et al. [39] first reported robot-assisted surgery for ECIPAS, there have been 3 cases including our case. Robot-assisted surgery has the advantages of less blood loss, fewer complications, less postoperative pain, faster recovery, and shorter hospital stay compared with laparoscopic and open distal pancreatectomy [48]. In addition, there are many reports of spleen preservation in robot-assisted surgery. It is thought that this is because a more delicate operation is possible because of the stereoscopic view and the stable forceps in addition to the magnifying effect of laparoscopic surgery. Robot-assisted surgery could be an effective option for distal pancreatectomy.

We report a relatively rare case of a patient with ECIPAS that was resected with robot-assisted surgery, and who showed good progress postoperatively, and we conducted a systematic review of the reported cases. Diagnosis is difficult, diagnostic treatment is often performed, and minimally invasive surgery is desired. Among minimally invasive surgical methods, robot-assisted surgery, which has been developed in recent years, appears to be a good option.

## Data Availability

The data and supplementary data are available from the corresponding author upon a reasonable request**.**

## Abbreviations

ECIPAS: Epithelial cyst in an intrapancreatic accessory spleen
US: Ultrasonography
CT: Computed tomography
MRI: Magnetic resonance imaging
EUS: Endoscopic ultrasonography
MCN: Mucinous cystic neoplasms
EUS-FNA: Endoscopic ultrasound-fine needle aspiration
